# Radiological and mid- to long-term patient-reported outcome after stabilization of traumatic thoraco-lumbar spinal fractures using an expandable vertebral body replacement implant

**DOI:** 10.1186/s12891-021-04585-y

**Published:** 2021-08-30

**Authors:** Siegmund Lang, Carsten Neumann, Christina Schwaiger, Andreas Voss, Volker Alt, Markus Loibl, Maximilian Kerschbaum

**Affiliations:** 1 Clinic of Trauma Surgery, University Medical Center Regensburg, Franz-Josef-Strauss-Allee 11, 93053 Regensburg, Germany; 2 Schulthess Clinic Zurich, Lenghalde 2, 8008 Zurich, Switzerland

**Keywords:** Thoraco-lumbar spinal fractures, Expandable vertebral body replacement, Posterior-anterior stabilization, Bony fusion rate, Patient-reported outcome measures, Health related quality of life

## Abstract

**Background:**

For the treatment of unstable thoraco-lumbar burst fractures, a combined posterior and anterior stabilization instead of a posterior-only instrumentation is recommend in the current literature due to the instability of the anterior column. Data on restoring the bi-segmental kyphotic endplate angle (BKA) with expandable vertebral body replacements (VBR) and on the mid- to long-term patient-reported outcome measures (PROM) is sparse.

**Methods:**

A retrospective cohort study of patients with traumatic thoraco-lumbar spinal fractures treated with an expandable VBR implant (Obelisc™, Ulrich Medical, Germany) between 2001 and 2015 was conducted. Patient and treatment characteristics were evaluated retrospectively. Radiological data acquisition was completed pre- and postoperatively, 6 months and at least 2 years after the VBR surgery. The BKA was measured and fusion-rates were assessed. The SF-36, EQ-5D and ODI questionnaires were evaluated prospectively.

**Results:**

Ninety-six patients (25 female, 71 male; age: 46.1 ± 12.8 years) were included in the study. An AO Type A4 fracture was seen in 80/96 cases (83.3%). Seventy-three fractures (76.0%) were located at the lumbar spine. Intraoperative reduction of the BKA in *n* = 96 patients was 10.5 ± 9.4° (*p* < 0.01). A loss of correction of 1.0 ± 2.8° at the first follow-up (t1) and of 2.4 ± 4.0° at the second follow-up (t2) was measured (each *p* < 0.05). The bony fusion rate was 97.9%. The total revision rate was 4.2%. Fifty-one patients (53.1% of included patients; age: 48.9 ± 12.4 years) completed the PROM questionnaires after 106.4 ± 44.3 months and therefore were assigned to the respondent group. The mean ODI score was 28.2 ± 18.3%, the mean EQ-5D VAS reached 60.7 ± 4.1 points. Stratified SF-36 results (ISS < and ≥ 16) were lower compared to a reference population.

**Conclusion:**

The treatment of traumatic thoraco-lumbar fractures with an expandable VBR implant lead to a high rate of bony fusion. A significant correction of the BKA could be achieved and no clinically relevant loss of reduction occurred during the follow-up. Even though health related quality of life did not reach the normative population values, overall satisfactory results were reported.

## Background

Spinal column injuries make up a relevant share in trauma patients. The thoracic and lumbar spine is frequently affected [1, 2]. A combined dorso-ventral stabilization has been shown to be a feasible procedure that can provide more stability for unstable traumatic vertebral fractures than a posterior- or anterior-only approach [3–5]. Additional to the posterior stabilization with an internal fixator, different methods for the restoration of the anterior column have been established, including the use of bone grafts and metal-mesh cages. Not least because tricortical bone grafts are associated with donor site morbidity [6, 7], vertebral body replacement implants (VBR), that can be implanted through a minimal-invasive surgical approach have been developed over the last decades, providing an individual anatomical adaptation [8]. Expandable implants have been proven to be an alternative to conventional cages and bone blocks [9–12]. Although their effectiveness in providing primary stability has been shown, data on restoring the bi-segmental kyphotic endplate angle (BKA) is still sparse. Noteworthy, the loss of ventral support and cage subsidence over time has been reported [13, 14]. It must be considered that a potential resulting kyphotic malalignment can impair spinal function and may even result in secondary neurological symptoms and disorders [15, 16]. Since several years the role of patient-reported outcome measure (PROM) has been emerging in trauma surgery, but the mid- to long-term outcomes after VBR implantation remain to be described more closely.

This study aimed to analyze the radiological and mid- to long-term PROM results after dorso-ventral stabilization of traumatic thoracic and lumbar spinal fractures using an expandable VBR (Obelisc™, Ulrich Medical, Ulm, Germany) for the reconstruction of the anterior column in a cohort of 96 patients.

## Methods

### Inclusion and exclusion criteria

Patients with traumatic thoraco-lumbar spinal fractures treated with a VBR implant in our trauma department (level 1 trauma center) between 01/2001 and 01/2015 were retrospectively identified for this study. Only patients with two radiological follow-ups were included, of which the second was conducted at least 24 months after the VBR surgery. Patients younger than 18 and older than 69 years and patients with pathologic fractures and with the diagnosis of osteoporosis, that has previously been verified by dual energy x-ray absorptiometry (DXA), were excluded. Besides patient related data (sex, age, BMI), injury mechanism, additional injuries, Injury Severity Score (ISS), pre- and postoperative neurological status according to the American Spinal Injury Association Score (ASIA), fracture type as well as treatment details (surgical regime; surgery time; time between surgeries; hospitalization time; time on the intensive care unit (ICU)), complications and revision surgeries were evaluated retrospectively based on the patients’ records. PROMs were collected and evaluated prospectively.

### Ethics approval and consent to participate

This study was carried out in accordance with the Declaration of Helsinki and approved by the ethics committee at the University of Regensburg in 03/2017 (Institutional Review Board Number 2017–0781-10). Written informed consent was obtained from all individual participants included in the study.

### Fracture classification and surgical procedure

Fractures were classified based on plain X-rays and computer tomographic (CT) scans according the AOSpine thoraco-lumbar injury classification system [17]. Indications for the reconstruction of the ventral column were an AO Spine Type A2 with wide fracture separation and/or relevant lesion of the intervertebral disc and A4 vertebral fractures with considerable deviation in the endplate angle [18]. Type A3 fractures with insufficient ventral stability depending on the destruction of the vertebral body were also considered for the reconstruction of the anterior column. Further general indications for the surgical strategy were a critical narrowing of the spinal canal and considerable deviation in the endplate angle and/or the scoliosis angle. All patients were treated by one senior spine surgeon (CN) or under his supervision with a single type of expandable titanium cage for vertebral body replacement (VBR) (Obelisc™, Ulrich Medical, Ulm, Germany). For the treatment of fractures of the thoracic spine, down to L2 fractures a thoracoscopic-assisted surgery approach was used [19] and for lumbar fractures a minimally invasive ventral/retroperitoneal approach was used. While performing discectomy of the cranial and caudal intervertebral disc the adjacent endplates were kept intact. To stimulate the bony fusion, corpectomy bone was applied around the VBR implant. Prior to the vertebral body replacement, dorsal instrumentation, anatomic reduction, and stabilization with a minimal invasive, fixateur interne system (three different systems were used: uCentum™ and tangoRS™, both Ulrich Medical, Ulm, Germany; USS MIS Fracture System, DePuy Synthes, Raynham, MA, U.S.) was performed in prone position. Apart from the Schanz Screw system (USS MIS Fracture System, DePuy Synthes) no mono-axial (pedicle-)screws or index screws were used. As an alternative to Schanz Screws pre-fixation screws were chosen (uCentum™, Ulrich Medical), if dorsal fracture reposition was indicated. In cases, in which fracture reposition could be achieved by patient positioning and external reposition maneuvers polyaxial screws were used. The ventral stabilization was either achieved in the same session or as part of a sequential two-step procedure.

### Radiological assessment

**Fig. 1:**
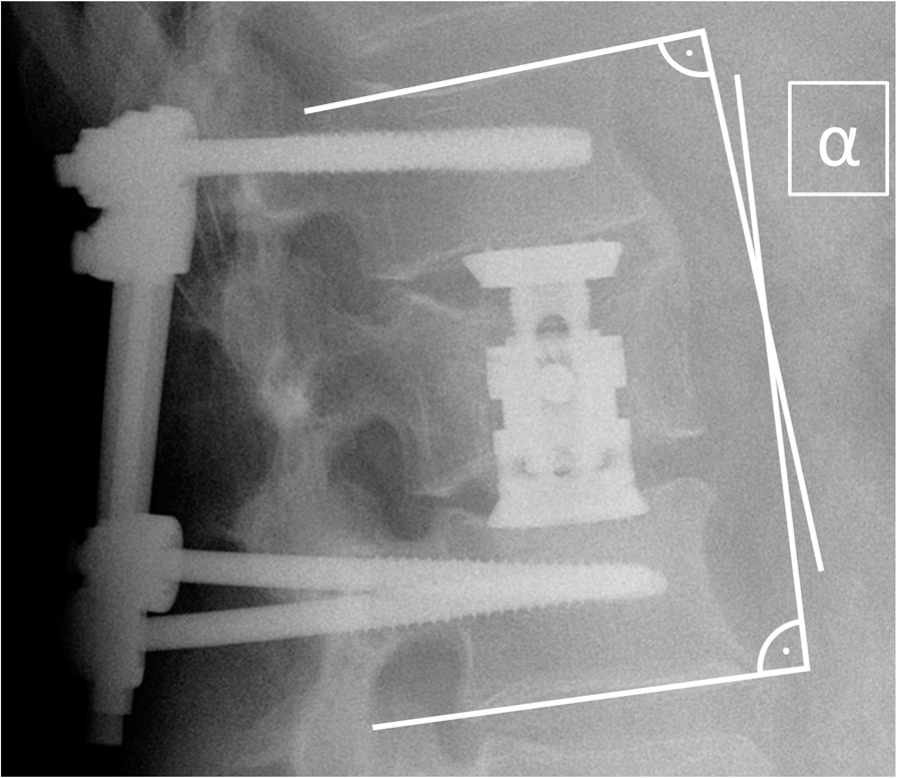
Measurement of the bisegmental kyphotic endplate angle α between a line drawn parallel to the superior endplate of one vertebra above the fracture, and a line drawn parallel to the inferior endplate of the vertebra below the fracture

All patients received pre- and postoperative CT scans and plain X-rays for surgery planning and verification of the correct implant position. Postoperative X-rays mark the time point t0. Additionally, plain X-rays were taken 6 months (t1) and at least 24 months after the VBR surgery (t2). Plain, lateral X-rays were used to measure the bi-segmental kyphotic endplate angle (BKA) (Fig. 1) [20]. Fusion rates were assessed in the lateral X-rays by evaluating absence of radiolucencies, absence of bone sclerosis and evidence of bridging trabecular bone within the fusion area. Further, if signs of screw loosening or implant displacement were seen, pseudarthrosis was assumed. Negative BKA values indicate kyphosis and positive values lordosis. All measurements were performed digitally using the software package OsiriX MD (Pixmeo, Bernex, Switzerland).

### Health related quality of life (QoL)-instruments

The German Short-Form 36 (SF-36) [21], Oswestry Disability Index (ODI) [22, 23] and EuroQol in 5 Dimensions (EQ-5D) were collected prospectively. All included patients (*n* = 96) were contacted by telephone and asked to participate in the study. If patients were not reachable by phone, forms were sent to the last known address. As a reference, normative data from Germany was used on the SF-36 [24] and EQ-5D [25]. For the SF-36, the physical components summary (PCS) and mental components summary (MCS) were calculated as described by Ellert et al. [24]. We assumed “minimal” and “moderate disability” in the ODI and “excellent” and “moderate” scoring in the EQ-5D subdimension to be “satisfactory”.

### Statistics

Statistical analysis was carried out using SPSS software version 24 (SPSS Inc., Chicago, Illinois). The Kolmogorov-Smirnov test was used to test for normality of the variables. Chi squared test was used for the comparison of categorical, nominal data. The Mann-Whitney U and Kruskal-Wallis tests were performed to compare categorical, ordinal variables for two or more than two groups respectively. The independent t-test was used to compare continuous variables after determining the distribution was appropriate for parametric testing. *P*-values < 0.05 were considered significant. Data is presented as mean ± standard deviation (SD) for continuous variables and as absolute and relative frequencies for categorical data.

## Results

**Fig. 2:**
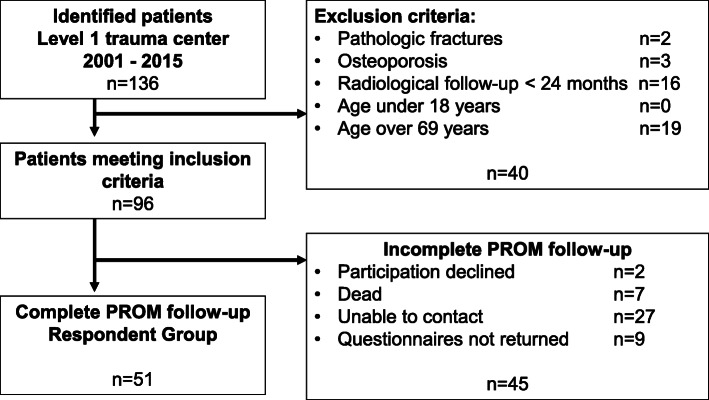
Flowchart: From 136 patients treated between 2001 and 2015 *n* = 96 met the inclusion criteria. Fifty-one patients completed the PROM follow-up and therefore were assigned to the respondent group

After the initial screening of the data base, *n* = 96 (25 female, 71 male) patients met the inclusion criteria for radiological evaluation (Fig. 2). The mean age was 46.1 ± 12.8 (19–69) years. The mean time to the first follow-up was 21.7 ± 19.1 (6–69) months and 49.8 ± 46.0 (24–213) to the second radiological follow-up.

Fifty-one patients (11 female, 40 male) with complete radiological follow-up completed the PROM questionnaires and therefore were defined as “respondent group”. All patients without a complete PROM follow-up were defined as non-responders, resulting in a loss to (PROM-) follow-up of 46.9%. The mean age of the patients in the respondent group was 48.9 ± 12.4 years, the mean BMI was 25.6 ± 4.1 kg/m^2^. The mean age of the patients that were lost to follow-up was 43.3 ± 12.5 years and the mean BMI was 24.5 ± 3.6 kg/m^2^. Patients lost to follow-up were significantly younger than patients in the respondent group (*p* < 0.05), but there was no difference in the BMI of the compared groups. There was no statistically significant difference in the fracture localization, the morphology of the vertebral body fracture or the presence of an additional C-classified injury between the respondent and non-respondent group (all *p* > 0.05) In the respondent group the first radiological follow-up was conducted 11.1 ± 5.1 (6-25) months after index surgery, the second after 42.1 ± 38.8 (24–181) months. The mean time between index surgery and completion of the PROM questionnaire was 106.4 ± 44.3 (26–179) months.

### Trauma mechanism, fracture classification and neurological symptoms

The most common fracture in the included patients was classified as AO Type A4 (*n* = 80; 83.3%). AO-Type A3 fractures were seen in *n* = 9 patients (9.4%) and AO-Type A2 in *n* = 7 patients (7.3%). An additional AO Type C injury was found in *n* = 26 (27.1%) cases. The most frequently affected vertebral body was L1 (26.0%). In *n* = 23 (24.0%) cases the fracture was in the thoracic spine, in *n* = 73 (76.0%) in the lumbar spine. Of these, *n* = 59 (61.5%) fractures were located at the thoraco-lumbar transition (Th11 – L2, overlapping with thoracic and lumbar fractures). Table 1 provides an overview of all fracture locations (thoracic, thoraco-lumbar, or lumbar) and differs between all included patients (*n* = 96) and the respondent group (*n* = 51). Ninety (93.8%) patients suffered from a bi-segmental fracture (considering A2, A4 and C injuries) and *n* = 6 (6.3%) suffered from a mono-segmental injury.

**Table 1:**
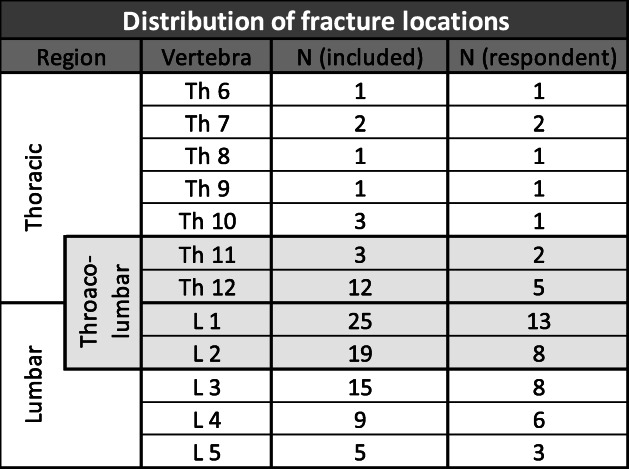
Distribution of fracture locations

Distinction between thoracic and lumbar spine. In all included patients (*n* = 96) *n* = 23 fractures were seen at the thoracic (Th6-Th12) and *n* = 73 fractures at the lumbar spine (L1-L5). Of these, *n* = 59 fractures were located at the thoracolumbar transition (Th11-L2). Accordingly, in the respondent group *n* = 13 fractures were located at the thoracic, *n* = 38 at the lumbar and of these *n* = 28 at the thoracolumbar transition

Preoperatively, *n* = 22 (22.9%) patients suffered from neurological symptoms. In detail *n* = 74 (77.1%) patients had normal neurological function (ASIA E). Fifteen (15.6%) patients showed incomplete deficits (ASIA B, C, and D) and seven (7.3%) patients had a complete deficit (ASIA A). Postoperatively, complete deficits persisted in four patients and three patients with incomplete deficits showed no improvement. Two ASIA A patients improved to each one ASIA B and one ASIA D respectively. Two ASIA B patients improved to each one ASIA C and one ASIA E respectively. Three ASIA C patients improved to ASIA D and one ASIA C patient improved to ASIA E. Five ASIA D patients improved to ASIA E. No new neurological deficits were reported postoperatively. In two cases, postoperative documentation did not include the ASIA scores or a detailed description of neurological symptoms. In the respondent group complete neurological deficits (ASIA A) persisted postoperatively in three patients. Three patients reported incomplete neurological deficits (ASIA B, C or D) postoperatively and another three patients improved from ASIA D to ASIA E.

Initially three (3.1%) patients could not be examined regarding the neurological status due to unconsciousness. From the three initial unconscious patients none reported neurological impairment postoperatively.

In the respondent group *n* = 22 (43.1%) patients suffered a traffic accident, nine (17.6%) had a fall from a height more than three meters and *n* = 10 (19.6%) from less than three meters. In *n* = 10 (19.6%) cases the trauma mechanism could not be extracted from the patients’ records. In the respondent group *n* = 39 (76.5%) patients had additional injuries including additional spine injuries (Table 2). The mean ISS in the respondent group was 11.8 ± 5.4 and *n* = 9 (17.6%) patients were characterized as “polytrauma” patients with an ISS ≥ 16.

**Table 2:** Additional injuries

	N (N_included_ = 96)	%	N (N_respondent_ = 51)	%	***P***
**None**	**24**	**25.0**	**12**	**23.5**	
**Additional injuries**	**72**	**75.0**	**39**	**76.5**	0.73
Extremities	34	35.4	14	27.5	0.07
Abdomen	5	5.2	2	3.9	0.53
Thorax	27	28.1	16	31.4	0.50
Pelvis	8	8.3	2	3.9	0.09
Spine	35	36.4	19	37.3	0.93
Head/Face	6	6.3	4	7.8	0.51

Comparison between included patients (*n* = 96) and the respondent group (*n* = 51). There was no statistically significant difference in the frequency of additional injuries detected between the included patients and the respondent group

### Treatment

All patients, included in this study, were treated operatively with a VBR device. All patients received posterior instrumentation: 78 patients (81.3%) received bi-segmental instrumentation. Due to high grades of instability, in *n* = 18 (18.8%) cases longer posterior constructs, up to four segments were chosen. Posterior decompression was performed in *n* = 43 (44.8%) patients, posterior spondylodesis in *n* = 51 (53.1%) patients. Sixty-seven (69.8%) patients were treated with a two-step sequenced procedure and the mean time between surgeries was 9.1 ± 5.6 days. The VBR surgery was conducted 8.1 ± 6.5 days after admission to our hospital including one- and two-step procedures. The mean surgery time for the VBR implantation was 115.8 ± 35.4 min in the two-step procedure. The mean surgery time for a one-step procedure was 174.6 ± 65.7 min. In the respondent group a thoracoscopic approach was chosen in *n* = 26 (51.0%) patients. In *n* = 25 (49.0%) patients a ventral/retroperitoneal approach was performed. Between the different surgical approaches, no statistically significant difference in the surgery duration was detected (*p* = 0.52). In the respondent group posterior decompression was performed in *n* = 11 (21.6%) patients and posterior fusion in *n* = 32 (62.7%) patients. All patients in the respondent group received a VBR (Obelisc™, Ulrich Medical, Ulm, Germany). In six of 51 (11.8%) cases an additional anterior angular stable plate system (goldengate®, Ulrich Medical, Ulm, Germany) was used. In those cases, sufficient ventral stabilization found to be in doubt because of poor bone quality and so the implantation of an additional anterior plate was indicated intraoperatively.

### Complications

Twenty-four (25.0%) patients were admitted to the intensive care unit (ICU) after the VBR surgery. The mean stay at the ICU was 5.8 ± 7.1 days. However, *n* = 15 (15.6%) patients were immediately admitted to the ICU after assessment at the emergency room due to general, severe injury. The overall complication rate was 10.4% (10/96): Four (4.2%) patients developed a surgical site infection, from which two (2.1%) were classified as superficial infections and two (2.1%) as deep infections. Three (3.1%) patients suffered prolonged wound healing with wound secretion or a liquor fistula in one case, which was treated with a liquor drain for 10 days. Afterwards no residual fistula occurred. In three (3.1%) cases implant loosening or malposition was recorded. In two (2.1%) patients, insufficient fusion was seen during the follow-up including one patient with signs of loosening of the dorsal instrumentation. Yet, those patients were free of complaints and no signs of instability were seen, so no revision surgery was conducted for those patients. Insufficient fusion without signs of implant loosening was not accounted as a complication.

The total revision rate was 4.2% (4/96) and all revisions occurred within the respondent group (7.8%): All patients in the respondent group with deep infection (*n* = 2) and screw or Golden Gate malposition (*n* = 2) had to be revised: The two infection cases were classified as acute infections of the dorsal instrumentation, within 6 weeks after the index surgery. The VBR implant has not been affected by the infection. Debridement and implant retainment of the dorsal instrumentation was performed in both cases, followed by an antibiotic treatment regime including biofilm active antibiotics.

Two (2.1%) patients, in whom malposition of the ventral plate or the dorsal instrumentation was detected in the postoperative CT-Scan were revised immediately and the implants were replaced. In no case a revision of the VBR was indicated. No statistically significant difference in the occurrence of complications was detected between the different surgical approaches (*p* = 0.91). Altogether, complications were recorded in *n* = 6 cases (11.8%) in the respondent group. No in-hospital deaths were documented.

## Radiological outcome

### BKA of the included patients (*n* = 96)

**Fig. 3:**
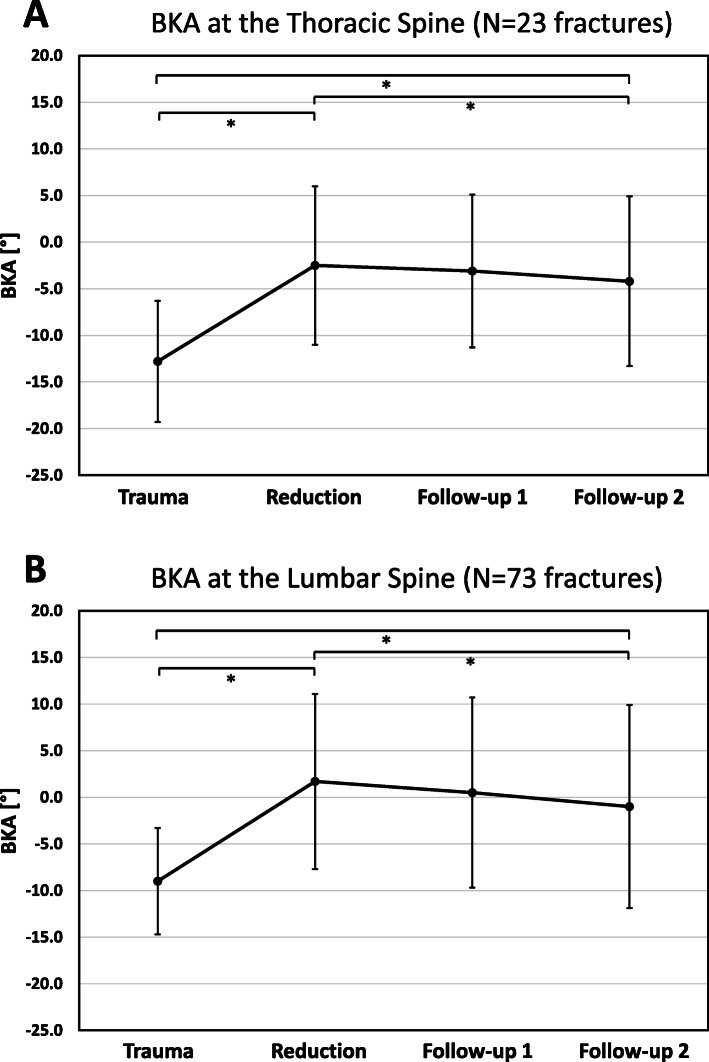
Visualization of the changes in the BKA (96 included patients) of the thoracic and lumbar spine. The time to the radiological follow-up at the thoracic spine were: Follow-up 1 (t1): 14.4 ± 13.9 months and follow-up 2 (t2): 41.8 ± 36.7 months; The time to the radiological follow-up at the lumbar spine was: Follow-up 1 (t1): 22.9 ± 19.3 months and follow-up 2 (t2): 52.6 ± 48.8 months. At the thoracic spine (**A**; 23 patients) a mean preoperative BKA of − 12.8° ± 6.5° could be significantly corrected by 10.3° (reduction) and a statistically significant loss of reduction during the follow-up by − 1.7° (t2) was observed. At the lumbar spine (**B**; 73 patients), a mean preoperative BKA -9.0° ± 5.7° could be significantly reduced by 10.7° (reduction) and a statistically significant loss of reduction during the follow-up by − 2.7°(t2) was observed. * indicates *p* < 0.05

Within all included patients (*n* = 96), an average preoperative BKA of − 9.9 ± 6.1° was found, if not differentiated by fracture location. Although the kyphosis angle could be significantly reduced by 10.5 ± 9.4° (t0) with surgery (*p* < 0.01), a significant loss of reduction of 1.0 ± 2.8° at the first follow-up (t1) and of 2.4 ± 4.0° at the second follow-up (t2) was measured (each *p* < 0.05). Figure 3 illustrates the development of the BKA at the thoracic (A) and the lumbar (B) spine. The fusion rate in the included patients was 97.9% (94/96) at the second radiological follow-up.

### BKA respondent group (*n* = 51)

Table 3 displays the changes in the BKA of the matched pairs of thoracic and lumbar fractures of the respondent group (*n* = 51), respectively. At the thoraco-lumbar transition surgery changed the BKA significantly by 9.6 ± 7.6° (*p* < 0.01). Loss of reduction was statistically significant with 1.7 ± 3.1° (t1; *p* < 0.01) and with 3.6 ± 4.5° (t2; *p* < 0.01). In the respondent group fusion was seen in 49 patients (96.1%) after the second follow-up (t2; 42.1 ± 38.8 months). Dorsal stabilization remained in *n* = 43 patients (84.3%) and was removed in eight patients (15.7%) after 13.1 ± 7.2 months in the respondent group. Of these, six patients initially suffered an incomplete burst fracture (AO Type A3). Two patients suffered implant irritation but showed complete fusion.

**Table 3:** Changes in the bi-segmental kyphotic endplate angle (respondent group)

	Time Points	Mean ± SD [°]	Range [°]	***P***
**Thoracic spine**	**Reduction**	−10.9 ± 6.9	−20.3 to 0.5	**< 0.01**
	t0 vs t1	0.4 ± 2.4	− 2.6 to 6.6	**0.523**
	t0 vs t2	1.9 ± 3.2	−2.9 to 7.8	**< 0.05**
**Lumbar spine**	**Reduction**	−9.1 ± 8.8	−27.5 to 5.9	**< 0.01**
	t0 vs t1	1.6 ± 3.0	−1.6 to 10.6	**< 0.01**
	t0 vs t2	3.0 ± 4.2	−2.9 to 15.3	**< 0.01**

Changes in the BKA between preoperative and postoperative (Reduction) and the different time points (t0, t1, t2) in the follow-up of the respondent group (*n* = 51). *P*-Values in bold indicate significant changes

### Patient-reported outcome

**Fig. 4:**
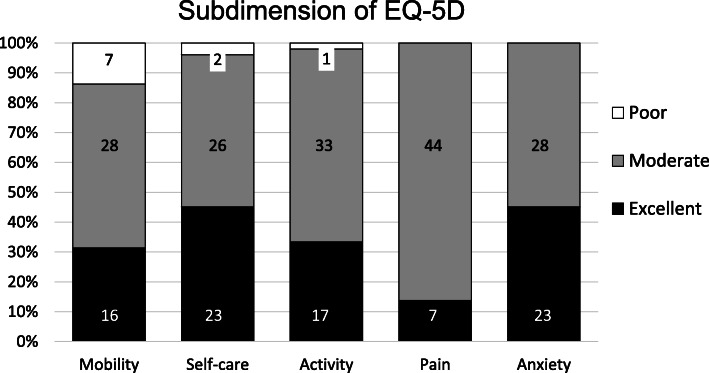
The five subdimension of EQ-5D. The columns represent 100% of patients and the absolute numbers in the columns show the distribution of outcomes in “Poor”, “Moderate” and “Excellent”

EQ-5D VAS reached 60.7 ± 4.1 points at the follow-up. No significant differences between the location of the fracture (thoracic/lumbar) were found (*p* = 0.59). Figure 4 displays the subdimensions of EQ-5D. The mean EQ-5D Index was 0.7 ± 0.3. Patients with a postoperative complete neurological deficit (ASIA A) reported a significant lower EQ-5D Index (0.1 ± 0.1) compared to the patients with incomplete (0.6 ± 0.2) or no neurological deficit (0.7 ± 0.2) (all *p* < 0.05). There was no statistically significant difference in the EQ-5D Index between patients with postoperative incomplete deficits (ASIA B, C, D) and no neurological deficits (ASIA E) (*p* = 0.76). Patients with multiple traumas or with an ISS ≥ 16 reported a significant lower EQ-5D Index than patients with a mono-trauma of the spine or an ISS < 16 (both *p* < 0.05).

**Fig. 5:**
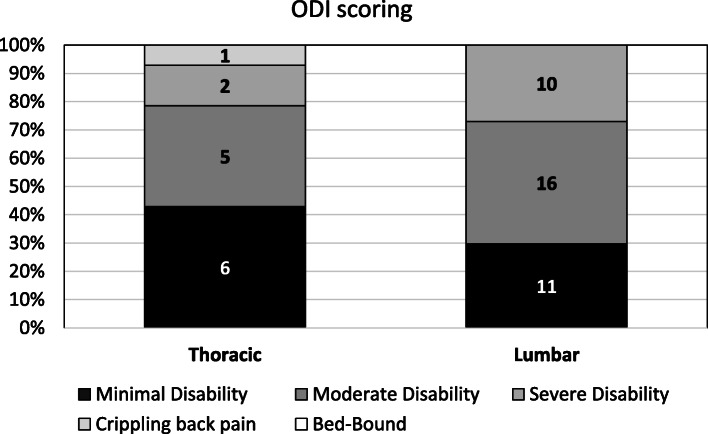
The five categories of disability in ODI scoring and the distribution of patients with surgery at the thoracic and the lumbar spine. The columns represent 100% of patients and the absolute numbers of cases are given within the respective category

The mean ODI score of the total respondent cohort was 28.2 ± 18.3% representing moderate impairment. Seventeen (33.3%) patients reported minimal, *n* = 21 (41.2%) moderate and *n* = 12 (23.5%) severe disability. One patient (2.0%) reported crippling back pain and no patient suffered exacerbated back pain or was bed-bound. There was no significant difference in the distribution of the scorings between thoracic and lumbar fractures (*p* = 0.50) (Fig. 5) or between patients with multiple or mono-trauma (*p* = 0.65). Further there was no difference in the ODI score depending on the ISS < or ≥ 16 (*p* = 0.76).

Table 4 shows the results of the eight main SF-36 items and the PCS and MCS of the respondent group in comparison to a healthy reference population [24]. The results were stratified according to ISS and *n* = 9 patients (17.6%) were classified as polytraumatized patients with an ISS ≥ 16. Both groups showed lower values compared to the healthy population for each item, independent from the ISS value. Polytraumatized patients (ISS ≥ 16) showed a trend to lower SF-36 values in all items and the PCS and MCS (Table 4). Comparing thoracic and lumbar location of the treated fracture or patients with multiple or mono-trauma, no significant difference between the single items was found (each *p* > 0.05). In further subgroup analyses we compared patients without any postoperative neurological deficit (ASIA E) and patients with mono-traumata with the healthy reference group and again detected lower values in all eight main SF-36 items compared to the normative values. No statistical difference in all PROM parameters was seen depending on removal of the metalwork (all *p* > 0.05).

**Table 4:** Main SF-36 items

Item	German reference population 2013 [24]	Study population (***n*** = 51)	
		ISS < 16 (***n*** = 42)	ISS ≥ 16 (***n*** = 9)
Physical functioning	89.5 (88.3–90.7)	57.7 ± 4.2	26.7 ± 8.9
Role physical	85.5 (84.1–86.9	51.2 ± 7.1	22.2 ± 14.7
Role emotional	86.8 (85.3–88.2)	57.7 ± 7.1	40.7 ± 16.5
Energy	60.7 (59.4–61.9)	49.0 ± 2.4	48.3 ± 10.3
Emotional Well-Being	72.8 (71.6–73.9)	65.0 ± 2.5	63.6 ± 7.3
Social functioning	85.6 (84.2–87.1)	74.4 ± 3.5	55.6 ± 9.6
Bodily Pain	75.3 (73.6–76.9)	62.4 ± 3.4	51.1 ± 12.5
General health	69.9 (68.8–71.1)	51.3 ± 3.4	53.7 ± 8.4
PCS	52.4 (51.9–52.9)	38.8 ± 1.8	33.4 ± 4.7
MCS	48.8 (49.1–49.5)	38.9 ± 2.6	30.8 ± 6.0

Stratified scores for injury severity (ISS < and ≥ 16): The eight main SF-36 items and the physical component score (PCS) and mental component score (MCS) in comparison to the normative data of the German population [24]. The reference values are presented as mean with 95% confident interval. There remains difference between the respondent group and a healthy German population in all main items, PCS and MCS

To assess the influence of loss of reduction on the PROM we defined relevant loss of reduction as a change of the BKA between the postoperative X-ray and the second follow-up by ±3.0°. In *n* = 11 (21.6%) cases of the respondent group a relevant loss of reduction of 8.8° ± 3.0° at average was found. In all other cases the mean change in the BKA was 1.0° ± 2.1°. There was no significant difference in the age of patients with significant loss of reduction (49.8 ± 9.9 years) and without significant loss of reduction (48.2 ± 13.4 years) (*p* = 0.70) or in the location of fractures at the thoracic (*n* = 2; 14.3%) or lumbar spine (*n* = 9; 24.3%) (*p* = 0.44). Between the two groups with and without a relevant loss of reduction no statistically significant differences in all SF-36 items, ODI and EQ-5D parameters were detected (all *p* > 0.05).

## Discussion

The use of an expandable vertebral body replacement implant showed to be a feasible procedure with only few implant-associated adverse events of the dorsal instrumentation and a revision rate of 4.2% in the current study population. A high bony fusion of 97.9% was seen, in concordance with the existing literature [10, 26]. All 96 patients underwent surgery with insertion of a single type of expandable vertebral body replacement implant and VBR implant dislocation was seen in no patient. Loosening or misplacement of pedicle screw was observed in only two patients. Unlike other studies, that included patients with heterogenous indications and examined different implants, the current homogenous cohort contains only trauma patients with a minimum radiological follow-up of 2 years and a mean PROM follow-up of 106 months.

The presence of additional injuries and the trauma severity (ISS ≥ 16) must be discussed as a possible confounder of the PROM result. Many patients (76.5%) in the respondent group suffered from additional injuries of one or more body regions and in three cases complete neurological deficit (ASIA A) persisted postoperatively. Only nine (17.6%) patients in the respondent group were classified as polytraumatized patients with an ISS ≥ 16. Patients with postoperative complete neurological deficits or an ISS ≥ 16 showed significant worse outcomes in the EQ-5D index and a trend to worse outcomes in the SF-35 and EQ-5D scores. McLain et al. documented in a 5-year follow-up after spinal injuries of 62 severely injured patients treated with segmental spinal instrumentation, that a neurological injury had a greater impact on functional outcome than other variables [15].

The results of this study showed, that health-related QoL in terms of the SF-36 could not return to the values of an age-matched reference population, regardless the trauma severity [24]. Even if patients without residual neurological deficits (ASIA E) were analyzed separately, the level of a healthy German reference group could not be reached in terms of all eight main SF-36 items. Neither severely injured patients (ISS ≥ 16) nor less severe injured patients (ISS < 16) regained normative population values (PCS 33.4 and 38.8 vs. 52.4; MCS 30.8 and 38.9 vs. 48.8). Smits et al. conducted a study on the anterior stabilization of thoraco-lumbar fractures and compared SF-36 scores at least 1 year after surgery with a cohort that underwent posterior fixation only in the same hospital and with values of the normative healthy population. In line with our results, they found that health-related QoL in their cohort did not return to that of the normative population, but was comparable to that of patients with less severe fractures [27]. They reported higher SF-36 component scores as well for severely injured patients (PCS = 40.0; MCS = 49.0) as for less severely injured patients (PCS = 47.0; MCS = 46.0) compared to our mid- to long-term health-related QoL results [27]. However, regardless the high number of additional injuries and neurological deficits, the overall functional outcomes in our study were found to be satisfactory in terms of the EQ-5D and ODI scores. The EQ-5D subdimensions show the distribution of cases with only a few “poor” results and mostly “moderate” or even “excellent” results. Regarding the ODI outcome only one patient reported crippling back pain and no patient suffered exacerbated back pain or was bed-bound. Consistent with our findings of an average EQ-5D index of 0.7, Deml et al. reported the same mean EQ-5D index of 0.7 at a 1.7-year follow-up after anterior column reconstruction of the thoraco-lumbar spine with a new modular PEEK vertebral body replacement device in 48 cases [28]. The mean ODI score reported in the current cohort was 28.4%, indicating moderate impairment (defined as 21.0–40.0%). Similar to this finding, Kumar et al. reported a mean ODI score of 14 points (=28.0%), 18 and 30 months after open surgery procedure for thoraco-lumbar burst fractures [29]. There was no evidence of neurological compromise in their collective and no additional injuries were reported, therefore representing a different collective with less severe injured patients. Schnake et al. reported an average ODI score of 12 points (=24.0%) at a five-year follow-up after posterior-anterior stabilization of traumatic thoraco-lumbar fractures using expandable titanium cages, which is also consistent with our results, nearly 9 years after surgery in average [10]. In line with our findings, Kreinest et al. reported most patients to be satisfied, 3 years after implantation of a hydraulic expandable VBR following traumatic thoracic and lumbar vertebral fractures [11]. Different to our methods they chose the VAS Spine score as an outcome parameter and found a mean rating of 65.2 ± 23.1 in their study population, which was found to be comparable to the rating of 58.4 from the German Spine Fracture Registry for operatively treated patients after spine fractures [4]. Comparable with our findings, they observed a significant reduction of functional outcome scores in their study cohort when compared to healthy subjects, but most of the patients (85.1%) were “generally/very satisfied” with their outcome [11]. Other study-groups like Spiegl et al. achieved better VAS Scores with a thoracoscopic procedure [30], but in the current study we could not identify a significant influence of the surgical approach or other surgery-related parameters on the health-related QoL.

In our respondent group all patients were treated with a combined anterior and posterior strategy and in only a few cases (15.7%) the dorsal metalwork was removed after 13 months at average. No statistical difference in all evaluated PROM parameters was seen depending on removal of the metalwork. The data found in literature does not suggest any difference in PROM depending on ventral or dorsal or combined surgery strategies of traumatic fractures of the thoracic and lumbar spinal column, but combined surgery is usually recommend for the treatment of instable thoraco-lumbar burst fractures due to an instable anterior column [4, 30, 31]. In a prospective cohort study, examining the six-year outcome of thoracoscopic ventral spondylodesis after unstable incomplete cranial burst fractures of the thoraco-lumbar transition Spiegl et al. compared ventral only and dorso-ventral treatment strategies and found a higher operative correction in dorso-ventrally treated patients [30]. They reported that a high percentage of the operative correction was lost after 6 years. Contrary, we did not see a clinically relevant loss of operative correction after a mean radiological follow-up of 42 months. Several authors claim an association between kyphotic malalignment and functional outcome [32, 33]. In contrast, other studies did not find a significant correlation between radiological and functional outcomes [4, 34]. In the current study *n* = 11 (21.6%) patients with a potential clinically significant loss of correction, which we defined as ±3.0° in the second follow-up were seen. Those patients did not report on worse health-related QoL results compare to patients with a stable correction. Noteworthy, the QoL outcome was assessed 106.4 ± 44.3 months after surgery, whereas the last radiological follow-up (t2) of the respondent group was conducted 42.1 ± 38.8 months after surgery. Therefore, the current results do not allow to evaluate a correlation between radiological and PROM results unconditionally.

The bony fusion rate in our cohort was 97.9% and in only two patients an a-symptomatic non-union was detected. No VBR implant had to be revised. The overall revision rate was 4.2%.

We showed a reduction of the BKA in *n* = 96 patients of 10.3° at the thoracic and of 10.7° at the lumbar spine (Fig. 3A & B) and similar values were found for the respondent group. At the thoraco-lumbar transition the BKA was significantly corrected by 9.6° in the respondent group. This is above the range of the reduction potential of posttraumatic kyphotic deformity, reported by the Spine Study Group of the German Trauma Association of 5.7° and 5.1° for the bi-segmental wedge angle using various implants with an open approach in the thoracic and lumbar spine, respectively [4]. In 2015 our study group conducted a clinical and radiological one-year follow-up assessment of 26 patient with fractures of the thoracic and lumbar spine treated with bi-segmental dorsal instrumentation with a minimal-invasive transpedicular Schanz Screw system [35]. For the reconstruction of the ventral column different methods were performed and an intraoperative correction of the BKA by 11.5° could be achieved. 5.8° loss of reduction was seen after 6 weeks. At the one-year follow-up a loss of reduction of 4.9° after dorsoventral stabilization was reported [35]. The current study reveals a loss of reduction of 2.7° at the lumbar spine (Fig. 3B) after approximately 3.5 years, which we estimate to be clinically irrelevant. At the thoracic spine, a statistically significant but clinical irrelevant loss of correction of 1.7° at the second follow-up was seen. In the respondent group a significant loss of reduction was seen during the follow-up, at the thoracic and the lumbar spine and at the thoraco-lumbar junction, which again was not estimated to be clinically relevant. It is worth noting, that the comparison between the pre- and postoperative angle includes a possible influence of patients positioning, as the pre- and postoperative CT scan was assessed in a supine position and follow-up X-rays were taken with the patient standing up-right [36]. This should be kept in mind not to overestimate the loss of reduction between the immediate postoperative BKA in supine position and the follow-up in up-right position.

Schnake et al. reported an average postoperative loss of correction of 2.4° due to minimal subsidence of the cages in a five-year clinical and radiological follow-up of 54 patients that had received combined anteroposterior stabilization of thoraco-lumbar fractures which is similar to the findings in our cohort [10]. Cage subsidence has been reported and was interpreted as a “settling down” of the anteroposterior construction, that appeared mainly in the first postoperative year [10]. Although not examined in detail, cage subsidence may have also played a role in the loss of reduction in our study. In 2020 Deml et al., who retrospectively reviewed 48 patients that underwent a corpectomy at heights between T5 and L5 due to trauma or tumor and were stabilized with the new PEEK vertebral body replacement showed, that the BKA was corrected by 12.1° at average [28]. At the end of 1.7 years follow-up, they observed a mean loss of correction of 1.6° and the fusion rate was 92.1% [28], which resembles our results.

Summarized, similar results regarding the current findings for intraoperative reduction (10.5°, all fractures), loss of reduction (2.4°, all fractures) and fusion rate (97.9%) can be found in the literature. Health related QoL oucomes in the current cohort approximately 9 years after VBR surgery regarding EQ-5D index (0.7) and ODI scoring (28.0%) were comparable to literature reported short- to mid-term results. Mid- to long-term SF-36 scores did not return to normative population values and are below literature reported short-to mid-term outcomes. Patients with persistent neurological impairment, additional trauma and an ISS ≥ 16 showed significantly lower EQ-5D indices but other PROM parameters were not significantly influenced.

### Limitations

This retrospective analysis has several strengths and limitations. The analyzed fractures in this large study group were all treated with the same surgical strategy using an expandable vertebral body replacement. The patients were followed for the QoL evaluation for a long time and it may be accounted to the length of the follow-up that there was a high loss to follow-up (46.9%). No relevant differences in patient and treatment parameters or in the distribution of fracture location or fracture morphology between the respondent group and the non-respondent group were seen.

The main limitation of this study is the lack of a control group and thus the lack of any randomization. For a definitive statement on the efficiency and safety of the use of the tested VBR implant for the reconstruction of the anterior vertebral column prospective randomized clinical trials will have to be conducted.

Although, patients with a DXA supported diagnosis of osteoporosis were excluded retrospectively not all patients were screened for osteoporosis before anterior-posterior stabilization. To minimalize potential confounding effects of osteoporosis we excluded all patients older than 69 years.

Furthermore, the respondent group includes patients with postoperative persistent complete and incomplete neurological deficits. Patients with a persistent complete neurological deficit (ASIA A) showed a significant worse EQ-5D index then patients with incomplete or with no neurological deficit. We presented additional stratified results for the QoL evaluation to put the severity of the trauma into perspective. Lastly, we presented a study with the limited cohort size of 51 patients in the respondent group. Statistical evaluation therefore must be interpreted with caution.

## Conclusion

The reconstruction and stabilization of traumatic unstable thoraco-lumbar spinal fractures with an expandable VBR implant has been shown to be feasible in the current study population. Further prospective studies will have to be conducted to prove the safety and efficiency of this procedure. A significant operative correction of the BKA was provided at both the thoracic and lumbar spine. No clinically relevant loss of correction was observed during the follow-up and a high bony consolidation rate of 97.9% was achieved. No revision surgery due to VBR dislocation was indicated. Satisfactory PROM results were found in a large part of the respondent group. However, quality of life did not reach the normative population values, regardless of trauma severity. Postoperative persistent neurological symptoms, additional trauma, and an ISS ≥ 16 were factors associated with a trend towards worse QoL outcomes.

## Data Availability

The datasets supporting the conclusions of this article are included within the article. Raw data can be requested from the corresponding author on reasonable request.

## Abbreviations

BKA: Bisegmental kyphotic endplate angle
BMI: Body mass index
CT: Computed tomography
DXA: Dual energy x-ray absorptiometry
EQ-5D: EuroQol in 5 Dimensions
ICBG: Iliac crest bone graft
ODI: Oswestry Disability Index
PROM: Patient reported outcome measure
QoL: Quality of Life
SD: Standard deviation
SF-36: Short Form 36
VAS: Visual analogue scale
VBR: Vertebral Body replacement

## References

[1] Katsuura Y, Osborn JM, Cason GW (2016). The epidemiology of thoracolumbar trauma: a meta-analysis. J Orthop.

[2] Smits AJ, den Ouden LP, Deunk J, Bloemers FW, Group LR (2020). Incidence of traumatic spinal fractures in the Netherlands: analysis of a Nationwide database. Spine.

[3] Wilke H-J, Kemmerich V, Claes LE, Arand M (2001). Combined anteroposterior spinal fixation provides superior stabilisation to a single anterior or posterior procedure. J Bone Joint Surg Br.

[4] Reinhold M, Knop C, Beisse R, Audigé L, Kandziora F, Pizanis A, Pranzl R, Gercek E, Schultheiss M, Weckbach A, Bühren V, Blauth M (2009). Operative treatment of traumatic fractures of the thoracic and lumbar spinal column: part III: follow up data. Unfallchirurg..

[5] Kim B-G, Dan J-M, Shin D-E (2015). Treatment of thoracolumbar fracture. Asian Spine J.

[6] Armaghani SJ, Even JL, Zern EK, Braly BA, Kang JD, Devin CJ (2016). The evaluation of donor site pain after harvest of tricortical anterior iliac crest bone graft for spinal surgery: a prospective study. Spine.

[7] Sheha ED, Meredith DS, Shifflett GD, Bjerke BT, Iyer S, Shue J, Nguyen J, Huang RC (2018). Postoperative pain following posterior iliac crest bone graft harvesting in spine surgery: a prospective, randomized trial. Spine J Off J North Am Spine Soc.

[8] Tarhan T, Froemel D, Rickert M, Rauschmann M, Fleege C (2015). History of vertebral body replacement. Unfallchirurg.

[9] Reinhold M, Schmölz W, Canto F, Krappinger D, Blauth M, Knop C (2007). Ein verbessertes Wirbelkörperersatzimplantat für die thorakolumbale Wirbelsäule: Biomechanischer In-vitro-Test an humanen Lendenwirbelkörpern. Unfallchirurg..

[10] Schnake KJ, Stavridis SI, Kandziora F (2014). Five-year clinical and radiological results of combined anteroposterior stabilization of thoracolumbar fractures. J Neurosurg Spine..

[11] Kreinest M, Schmahl D, Grützner PA, Matschke S (2017). Radiological results and clinical patient outcome after implantation of a hydraulic expandable vertebral body replacement following traumatic vertebral fractures in the thoracic and lumbar spine: A 3-year follow-up. Spine.

[12] Knop C, Blauth M, Bühren V, Arand M, Egbers HJ, Hax PM, Nothwang J, Oestern HJ, Pizanis A, Roth R, Weckbach A, Wentzensen A (2001). Surgical treatment of injuries of the thoracolumbar transition--3: follow-up examination. Results of a prospective multi-center study by the “spinal” study Group of the German Society of trauma surgery. Unfallchirurg..

[13] Schmieder K, Wolzik-Grossmann M, Pechlivanis I, Engelhardt M, Scholz M, Harders A (2006). Subsidence of the wing titanium cage after anterior cervical interbody fusion: 2-year follow-up study. J Neurosurg Spine.

[14] Briem D, Linhart W, Lehmann W, Bullinger M, Schoder V, Meenen NM, Windolf J, Rueger JM (2003). Investigation of the health-related quality of life after a dorso ventral stabilization of the thoracolumbar junction. Unfallchirurg..

[15] McLain RF (2004). Functional outcomes after surgery for spinal fractures: return to work and activity. Spine.

[16] Gertzbein SD (1994). Neurologic deterioration in patients with thoracic and lumbar fractures after admission to the hospital. Spine.

[17] Vaccaro AR, Oner C, Kepler CK, Dvorak M, Schnake K, Bellabarba C (2013). AOSpine thoracolumbar spine injury classification system: fracture description, neurological status, and key modifiers. Spine.

[18] Verheyden AP, Spiegl UJ, Ekkerlein H, Gercek E, Hauck S, Josten C, u. a (2018). Treatment of fractures of the thoracolumbar spine: recommendations of the spine section of the german society for orthopaedics and trauma (DGOU). Glob Spine J.

[19] Ringel F, Stoffel M, Stüer C, Totzek S, Meyer B (2008). Endoscopy-assisted approaches for anterior column reconstruction after pedicle screw fixation of acute traumatic thoracic and lumbar fractures. Neurosurgery.

[20] Keynan O, Fisher CG, Vaccaro A, Fehlings MG, Oner FC, Dietz J (2006). Radiographic measurement parameters in thoracolumbar fractures: a systematic review and consensus statement of the spine trauma study group. Spine.

[21] Bullinger M (1995). German translation and psychometric testing of the SF-36 Health Survey: preliminary results from the IQOLA Project. International Quality of Life Assessment. Soc Sci Med 1982.

[22] Roland M, Fairbank J (2000). The roland-morris disability questionnaire and the oswestry disability questionnaire. Spine.

[23] Fairbank JC, Couper J, Davies JB, O'Brien JP (1980). The Oswestry low back pain disability questionnaire. Physiotherapy..

[24] Ellert U, Kurth BM (2013). Health related quality of life in adults in Germany: results of the German health interview and examination survey for adults (DEGS1). Bundesgesundheitsblatt Gesundheitsforschung Gesundheitsschutz.

[25] Hinz A, Klaiberg A, Brähler E, König H-H (2006). The quality of life questionnaire EQ-5D: modelling and norm values for the general population. Psychother Psychosom Med Psychol.

[26] Heary RF, Kheterpal A, Mammis A, Kumar S (2011). Stackable carbon fiber cages for thoracolumbar interbody fusion after corpectomy: long-term outcome analysis. Neurosurgery..

[27] Smits AJ, Noor A, Bakker FC, Deunk J, Bloemers FW (2018). Thoracoscopic anterior stabilization for thoracolumbar fractures in patients without spinal cord injury: quality of life and long-term results. Eur Spine J Off Publ Eur Spine Soc Eur Spinal Deform Soc Eur Sect Cerv Spine Res Soc.

[28] Deml MC, Mazuret Sepulveda CA, Albers CE, Hoppe S, Bigdon SF, Häckel S, u. a (2020). Anterior column reconstruction of the thoracolumbar spine with a new modular PEEK vertebral body replacement device: retrospective clinical and radiologic cohort analysis of 48 cases with 1.7-years follow-up. Eur Spine J.

[29] Kumar A, Aujla R, Lee C (2015). The management of thoracolumbar burst fractures: a prospective study between conservative management, traditional open spinal surgery and minimally interventional spinal surgery. SpringerPlus..

[30] Spiegl U, Hauck S, Merkel P, Bühren V, Gonschorek O (2013). Six-year outcome of thoracoscopic ventral spondylodesis after unstable incomplete cranial burst fractures of the thoracolumbar transition: ventral versus dorso-ventral strategy. Int Orthop.

[31] Sasso RC, Renkens K, Hanson D, Reilly T, McGuire RA (2006). Unstable thoracolumbar burst fractures: anterior-only versus short-segment posterior fixation. J Spinal Disord Tech.

[32] Phan K, Nazareth A, Hussain AK, Dmytriw AA, Nambiar M, Nguyen D, Kerferd J, Phan S, Sutterlin C, Cho SK, Mobbs RJ (2018). Relationship between sagittal balance and adjacent segment disease in surgical treatment of degenerative lumbar spine disease: meta-analysis and implications for choice of fusion technique. Eur Spine J Off Publ Eur Spine Soc Eur Spinal Deform Soc Eur Sect Cerv Spine Res Soc..

[33] Domenicucci M, Preite R, Ramieri A, Ciappetta P, Delfini R, Romanini L (1996). Thoracolumbar fractures without neurosurgical involvement: surgical or conservative treatment?. J Neurosurg Sci.

[34] Wood K, Buttermann G, Butterman G, Mehbod A, Garvey T, Jhanjee R (2003). Operative compared with nonoperative treatment of a thoracolumbar burst fracture without neurological deficit. A prospective, randomized study. J Bone Joint Surg Am.

[35] Loibl M, Korsun M, Reiss J, Gueorguiev B, Nerlich M, Neumann C, Baumann F (2015). Spinal fracture reduction with a minimal-invasive transpedicular Schanz screw system: clinical and radiological one-year follow-up. Injury.

[36] Lee E, Woong Ko C, Suh W, Kumar S, Kyu K, Yang J-H (2014). The effect of age on sagittal plane profile of the lumbar spine according to standing, supine, and various sitting positions. J Orthop Surg.

